# 

**Published:** 1884-08

**Authors:** 


					﻿Vol. IV.
AUGUST, 1884.
NO. 8.
UHE
HOMEOPATHIC PHYSICIAN,
A MONTHLY JOURNAL OF MEDICAL SCIENCE.
“ Seek the Truth: come whence it may, cost what it will."
E. T. LEE, Js/L. ID.,
EDITOR.
CONTENTS:
(See inside of Cover.)
PHILADELPHIA:
No. 2109 CHESTNUT STREET.
NEW YORK:
A. L. CHATTERTON, PUBLISHING COMPANY, Publisher’s Agents.
LONDON:
ALFRED HEATH & CO., Sole Agents for Great Britain,
114 Ebury Street, Eaton Square.
'Yearly Subscription, $2.SO, invariably in advance.
Entered at the Philadelphia Post-Office as Second'Class Mail Matter.
				

